# Annotations

**Published:** 1888-06-09

**Authors:** 


### Annotations.

In a recent letter to the *Times*, Sir Edmund Hay Currie expressed the decided conviction that "all new hospitals should be started on provident lines." This is a wide and somewhat revolutionary generalisation, but Sir Edmund speaks with all the authority which belongs of right to long years of practical experience in hospital management. He states that "the Metropolitan Hospital in Kingsland Road was started on the 1st of November last year on provident principles, and that so far the results have been most satisfactory. The really poor who are unable to pay the regular fees of medical men pay regularly, sick or well, a small fixed monthly charge, and when ill can receive all the benefits which a general hospital affords, as well as the privilege of being visited, when necessary, at their own homes. That the poor appreciate this system, which prevents them from feeling that they are the recipients of charity, is proved by the large numbers which have joined the provident department. . . There are 6,000 lives on the books." Sir Edmund affirms "that new hospitals, by working on provident lines, prevent to a large extent the misuse of hospitals by persons who can well afford to pay a medical man; that they teach the poor to help themselves," and that "in time undoubtedly provident hospitals will be to large extent self-supporting." These opinions of a capable and experienced hospital manager must not be passed by as if they were the mere crude utterances of ignorance. Whatever be the dogmatisms and immovable prejudices of certain persons connected with hospitals, there is no doubt whatever that hospitals and all their ways are being slowly but surely dragged before the bar of a keen and enlightened public opinion. As to their work and the general principles of their management, they can well bear the fiery ordeal through which they may be called to pass. But to take up the position that no modification is, or ever can be, justifiable or desirable, is to undertake a defensive campaign which will require all the resources of skilled logic for its successful issue. At present we offer no opinion on the question. The 20th of June is approaching, when Mr. Burdett-Coutts is again to take the field at St. Thomas' Hospital. It may be hoped that in the interests of the huge and costly voluntary hospital system the discussion will be thoroughly statesmanlike, and worthy of the subject. As voluntary institutions hospitals are bound to make some attempt to satisfy the reasonable demands of their non-professional supporters. If they do not do this it is as certain as anything can be that voluntary support will gradually come to an end.

**Hospitals on Provident Principles.**

A sick bachelor is one of the most pitiable objects in the universe. Men are not the best of patients at any time. They do not take illness as one of the inevitable facts of life, to be borne without unnecessary grumbling, as women do; they look upon every headache as an evidence of special malignity on the part of fate, and the most tender care a wife can give will not make them otherwise than indignant, unreasonable, and impatient. But the sick bachelor—think of him! Alone in his comfortless lodging, dependent on the caprices of a landlady who thinks her life is a burden if the helpless invalid declines to consume a greasy, leathery chop and asks for beef-tea, which when it is brought is almost invariably a substance composed apparently of almost equal parts of grease and water, neither appetising nor nutritious. Poor bachelor! *His* pillow is not smoothed frequently by gentle hands, no cooling drinks of pleasant flavour are held to his lips, no fragrant perfumes are sprinkled over his brow, no murmured assurances that he is a "poor darling," no anxious questions, "Are you better now, dear?" fall on his lonely ear. His blind is not drawn to exclude the sun, nor his window opened to admit the air. He lies in his dreary room helpless and feverish, lonely and miserable, conscious that the only anxiety those around him feel about him is that he may turn out "an infectious case." Let him get a nurse, people say; she will give him all that training, if not love, can teach. But, alas! our bachelor is probably a poor one; he would not, it may be, remain a bachelor if he were otherwise, and the cost of a nurse, to be paid and kept by him at a time when he is earning nothing himself, is out of the question. Yet he would gladly pay a moderate sum to any competent, kindly woman who would come in, like the doctor, but more frequently and for longer visits, to make his bed with skilled hands, and leave him with all he will require in her absence near at hand. Is there not a need for a society that would provide nurses of this kind? In our poor districts there are nursing associations whose members go out to the sick of a whole district, going from one home to another with their comforting touch. These associations are purely charitable. Would it not, however, be possible to adapt the principle to patients who were able to pay a little for attendance, although they cannot afford the cost of the exclusive care of a trained nurse. There is certainly room for such an association, and all the poorer middle class would learn to avail themselves of it, as well as the bachelors, whose hapless case we have depicted.

**The Nursing of Bachelors.**

All lovers of "unity, peace, and concord" will be pleased at the successful opening of the Irish Exhibition at Olympia. Men, like dogs, when placed well apart and hounded and excited, are apt to think each other desperate monsters for whom nothing but extirpation is at all adequate punishment. If by any means they can be brought together on peaceful terms and made to look at each other patiently and quietly, illusions and delusions often disappear with much promptitude, and opposing parties come to think each other "not such very bad fellows after all." Irish industries have suffered from prolonged depression; Irish stomachs are as a consequence in some cases ill-satisfied, and Irish backs not seldom ill-clothed. Ireland has unique facilities for woollen manufactures; but it is stated that throughout the whole country there are not more than half-a-dozen prosperous mills. The machinery in use is in many cases out of date, and orders are few and small. It is hoped by many that the Exhibition at Kensington will give a new impetus, not only to the woollen manufactures but to other branches of Irish industry. Every countryman knows that for years Ireland has reared vast numbers of hardy and in many cases high-class horses. Similarly, Irish dairying is of universal fame. Canon Bagot, a Protestant parson, who to his ecclesiastical functions adds the part of poet, and king of the butter market, declared with the confidence of absolute conviction that the Kerry cows and the Kerry dairymaids are the handsomest in the world. No butter, he affirmed, either in this or any other universe could beat the genuine Irish article. The Baroness Burdett-Coutts and Mr. Burdett-Coutts, M.P., who have taken a deep interest in the Exhibition, have had brought over, almost entirely at their own expense, a number of boys from the great Baltimore fishery in county Cork. Mr. Ernest Hart, one of the most active members of the Executive Council, and a staunch friend of Ireland, explained the distinct objects of the Exhibition, and the Earl of Leitrim publicly recognised the cordial assistance received by the promoters from the directors of Olympia. It now only remains for the public to do their part by going to see the sights provided for their pleasure and instruction. That they will do so few who have seen the Exhibition can doubt. Indeed, the attendance is likely to be so large, as the interesting features of the Exhibition become known, that we counsel our readers to take an early opportunity of enjoying its many pleasurable features.

**The Irish Exhibition.**

